# Effects of needs-based education for prenatal anxiety in advanced multiparas: a randomized controlled trial

**DOI:** 10.1186/s12884-022-04620-3

**Published:** 2022-04-08

**Authors:** Quan Shen, Can-Ran Huang, Liu Rong, Shan Ju, Sharon R. Redding, Yan-Qiong Ouyang, Rong Wang

**Affiliations:** 1 School of Nursing, Wuhan University, No.115, Donghu Road, Wuchang District, Wuhan, 430071 China; 2 Project HOPE, Bethesda, MD 20814 USA; 3 Nursing Department of East Campus, Renming Hospital of Wuhan University, Gaoxin Sixth Road, Jiangxia District, Wuhan, 430071 China

**Keywords:** Needs-based education, prenatal anxiety, advanced multiparas

## Abstract

**Background:**

Prenatal anxiety is a common concern which may have adverse effects on maternal and infant health outcomes. Studies addressing needs-based education interventions for prenatal anxiety are limited.

**Aim:**

To explore the effects of needs-based education on alleviating prenatal anxiety among advanced multiparas when compared with routine prenatal health education.

**Methods:**

A total of 86 advanced multiparas were randomized into the intervention group (*n* = 43) or the control group (*n* = 43) in this study. The control group received routine prenatal care. The intervention group received five needs-based education programs presented by trained researchers. The Pregnancy-related Anxiety Questionnaire was used to evaluate changes in anxiety level of participants. Concurrent physiological parameters, including blood pressure, heart rate and non-stress test were also measured.

**Results:**

Scores on the Pregnancy-related Anxiety Questionnaire of the intervention group were significantly lower than those of the control group (*t* = 4.21, *P* < 0.05). Systolic blood pressure (*t* = 3.64, *P* < 0.05) and heart rate (*t* = 2.39, *P* < 0.05) of the intervention group were also significantly lower than the control group whereas no differences were noted in diastolic blood pressure and non-stress test.

**Conclusion:**

A needs-based education program is an effective intervention strategy to allay prenatal anxiety in advanced multiparas.

**Trial registration:**

The trial was retrospectively registered in the Chinese Clinical Trial Registry as number ChiCTR2100047552.

## Introduction

Prenatal anxiety is a common psychological disturbance during pregnancy. The current rate of prenatal anxiety was reported to be 20.7%, higher than 13.8% in the general population [1, 2]. Pregnant women with anxiety may present with clinical symptoms including agitation, cognitive distortions, constant worry, racing thoughts, shortness of breath, heart palpitations and restlessness [3]. In addition, prenatal anxiety affects maternal vasoconstriction accompanied by increased blood pressure (BP) and heart rate (HR), resulting in reduced blood supply to the placenta and a high probability of fetal distress in the uterus [4]. Also, prenatal anxiety increases the likelihood of preterm birth, low birth weight newborns and affects an infant’s emotional, neurological and cognitive development [5, 6].

A systematic review reported that the risk factors for poor gestational outcome related to prenatal anxiety included a previous history of mental illness, education level, employment or non-employed, income, and family support [7]. However, the relationships between parity or age and prenatal anxiety and depression were equivocal. Advanced multipara refers to pregnant women aged 35 years or older and having had at least one previous birth. With the implementation of the two-child policy in China, there is an increasing number of advanced multiparas [8]. Meanwhile, adverse pregnancy outcomes (e.g., cesarean section, preterm birth) have been reported to be associated with advanced maternal age and multi-parity, and anxiety during pregnancy may be related to adverse maternal and child health outcomes [9]. Therefore, advanced multiparas may present a higher level of anxiety, and interventions that alleviate prenatal anxiety in the vulnerable population are imperative to improve maternal and child health outcomes.

Globally, prenatal education has played an important role in improving perinatal psychological disorders [10, 11]. Although prenatal education in considered routine in many countries, it varies in content, quality and format [12]. A recent Cochrane qualitative review demonstrated that routine information in prenatal care is insufficient to satisfy women’s information needs [13]. Meanwhile, a cross-sectional study reported a significant association between patients’ perceived information needs and their anxiety level [14]. Therefore, providing needs-based prenatal education may be effective in reducing the anxiety level of pregnant women. Needs-based education involves assessing a patient’s need for information prior to providing content, which meets the goal of prenatal care and is the focus of patient-centered care [15]. Some randomized controlled trials (RCT) have reported the effectiveness of needs-based education on decreasing the anxiety level among surgery patients [16], and caregivers of patients in intensive care units [17]. However, evidence for using needs-based education to address perinatal anxiety remains limited and only suggestive. In light of this, the purpose of this study is to explore the effects of a needs-based education program on relieving prenatal anxiety of advanced multiparas, and it is also hypothesized that greater improvement of prenatal anxiety, as well as the anxiety-related physiological parameters in advanced multiparas following needs-based education compared with routine prenatal health education care.

## Methods

### Design

This was a RCT conducted at a prenatal clinic of a general hospital in a large city in central China. It was conducted from February to June 2021, and reported in accordance with the Consolidated Standards of Reporting Trials (CONSORT) statement [18].

### Participants

Purposive sampling was used to recruit participants. Inclusion criteria were as follows: (1) multiparous women; (2) aged 35 years or older; (3) at 26 ~ 28 weeks of gestation; (4) singleton pregnancy; (5) received prenatal care regularly; (6) able to read, write and communicate in Mandarin; and, (7) completed written informed consent. Exclusion criteria were: (1) women with pregnancy complications, acute disease, severe functional disorders or genetic disease; (2) women having preterm infants; and, (3) women experiencing major unexpected life events (e.g., divorce, serious illness or death of a partner).

### Sample size and randomization

Sample size was calculated a priori based on the G*Power software package (Version 3.1.4). At least 76 women (38 in each group) were required to detect the difference between two groups, with an effect size of 0.70 and a power of 85% at the 5% level of statistical significance. The effect size of 0.70 was estimated from a previous study examining the effects of prenatal intervention on pregnant women with anxiety [19]. Considering the anticipated 15% dropout rate, a total of 90 women were initially required to ensure an adequate final sample size. The actual post-hoc statistical power was calculated to be 89% in the current study.

Women included in this study were numbered (1–90) in the order of their prenatal visit, and a simple randomization method (1:1) was employed to assign each participant to either the intervention or control group, with 45 participants in each group. The allocation sequence was generated by the researcher using the SPSS software (Statistical Package of Social Sciences (SPSS), Version 23.0. Armonk, NY: IBM Corp).

### Procedure

#### Intervention group

The prenatal intervention program protocol targeting the patient’s knowledge needs in the current study was developed by conducting a systematic literature review, discussion with experts specializing in maternal and child health and talking with pregnant women. The protocol was submitted to a group of experts for review and confirmation. The group included three health professionals (obstetrician, midwife and nurse) who have been working in the obstetrics clinic for at least 10 years, and two professors who are specialized in nursing education employed at a nearby university for 15 and 20 years. The current prenatal intervention program was finalized after three revision and review sessions, and included five main topics focusing on knowledge on: (1) prenatal anxiety, (2) maternal age-related maternal and fetal risks, (3) psychological relaxation strategies, (4) use of medication during pregnancy and, (5) knowledge on labor and childbirth.

Participants in the intervention group received five 70-min prenatal education sessions. Each session was led by the same researchers, a nursing postgraduate student and an educator specializing in maternal and child health and included eight to ten participants. The group counseling sessions used a lecture format, and included case- and problem-based teaching strategies to ensure an interactive and effective learning environment. The outline of each session is presented in Table 1.

**Table 1:** Outline of the prenatal education intervention program

Session	Topic	Duration	Format	Teaching strategy
knowledge on prenatal anxiety	1. Welcome and orientation to the program 2. Introduction of prenatal anxiety 3. Adverse effects of prenatal anxiety 4. Discussion and feedback	60 min 10 min	Lecture	PowerPoint lecture Video presentation
Knowledge on maternal age-related maternal and fetal risks	1. Risk for mothers and fetus 2. Identification of abnormal symptoms 3. Risk prevention strategies 4. Case study 5. Discussion and feedback	60 min 10 min	Lecture	PowerPoint lecture, Video presentation, Case-based learning
Knowledge on psychological relaxation strategies	1. Psychological relaxation strategies 2. Progressive muscle relaxation training 3. Case study 4. Discussion and feedback	60 min 10 min	Lecture	PowerPoint lecture, Video presentation, Case-based learning
Knowledge on use of medication during pregnancy	1. Experience during medication uses 2. A short test on medication uses 3. Medication sensitivity during pregnancy 4. Medication information acquisition, understanding and evaluation 5. Case study 6. Discussion and feedback	60 min 10 min	Lecture	PowerPoint lecture, Video presentation, Case-based learning Problem-based learning
Knowledge on labor and childbirth	1. Indication of labor onset 2. Physical and psychological preparation for birth 3. Labor process and skills 4. Discussion and feedback 5. Summary of prenatal education program	40 min 10 min 10 min	Lecture	PowerPoint lecture, Video presentation,

Moreover, two to four individual counseling sessions (each 10–20 min in length) were also provided by researchers to each participant before the group education sessions started. The aim of the individual counseling was to deal with the confusion of each participant and provide the corresponding specific suggestions to them. The confusion varied among the participants, and the most common were the potential causes related to prenatal anxiety and how to assess or deal with it. The individual counseling was conducted using a question-and-answer format. The group and individual education sessions were interactive and contributors were fully involved in question and answer. Each education session was conducted in the classroom of the hospital. Considering that a majority of participants were employed, each session was conducted to coincide with a participant’s prenatal visit to the obstetrics clinic.

#### Control group

Participants in the control group received routine prenatal care at the prenatal clinic. Health education care was provided by the obstetrician during each prenatal visit, which included suggestions about healthy lifestyle (e.g., diet, exercise) in mid- and late pregnancy and preparing for admission with the onset of labor. Information was provided orally or in a hand-out. No information was provided regarding prenatal anxiety. At the end of the current study, participants in the control group were provided with educational material about prenatal anxiety and the opportunity for counseling.

### Measurements

#### Socio-demographic questionnaire

A researcher-designed demographic questionnaire was developed to obtain socio-demographic data on age, weeks of gestation, education level, monthly income, current residence, employment, only child or having siblings, gravidity, parity, previous delivery mode and planned/accidental pregnancy.

#### Pregnancy-related anxiety questionnaire

The Pregnancy-related Anxiety Questionnaire (PAQ) was employed to assess the presence and extent of anxiety among Chinese pregnant women [20]. It consists of 13 items with three subscales: anxiety of self (6 items), anxiety of child health (5 items) and anxiety of giving birth (2 items). Participants respond using a 4-point Likert scale from 1 (*never*) to 4 (*almost all the time*). Scores vary from 13 to 52, with a score of greater than or equal to 24 indicating pregnancy-related anxiety [21]. The Cronbach’s α was calculated to be 0.81 [20]. The Cronbach’s α was calculated to be 0.85 in the current study.

#### Physiological parameters

Blood pressure (BP) and heart rate (HR) were measured and record by an obstetrician using an electronic sphygmomanometer (HEM-770AFuzzy, OMRON, Japan). Participants were asked to rest quietly in a chair for at least 5 min before measuring BP and HR. The non-stress test (NST) was done by using an external monitoring device (MT-325, TOITU, Japan) over a period of 40 min, and the NST results were reviewed by the obstetrician. Participants were asked to relax in a semi-Fowler’s or left lateral position in a quiet environment. The results were evaluated following the criteria of the Chinese experts Ling and Gu [22]: reactive (+), reactive suspicious (±) and non-reactive (−).

### Data collection

Participants were contacted by face-to-face communication during their prenatal visit at the participating hospital. The PAQ was administered by a research assistant. Blood pressure, HR and the NST were also assessed by an obstetrician. After random allocation, the intervention group was assessed prior to the first and final education and counseling sessions. The control group was assessed at the first and final prenatal visits of participants after they were enrolled in the control group. The research assistant and obstetrician were blinded to the group allocation. Meanwhile, participants were asked to complete the socio-demographic questionnaires prior to the first education and counseling session in the intervention group and at the first prenatal visit after they were enrolled in the control group. This took approximately 10–15 min to complete.

### Data analyses

Data analysis was performed using the SPSS 23.0 (Statistical Package of Social Sciences (SPSS), Version 23.0. Armonk, NY: IBM Corp), with a significance level of 0.05 (two-tailed). Frequencies and proportions were used to display the categorical variables, and continuous variables were presented as mean and standard deviation. Two sample *t* test was used for continuous variables to compare the difference between the intervention and control groups. Paired sample *t* test was used for continuous variables to compare the difference between pre-test and post-test scores of participants in each group. Chi-square test and Fisher’s exact test were used for categorical variables to compare the difference between the two groups.

### Ethical considerations

All participants were asked to complete an informed consent and were assured that their responses would remain confidential and anonymous. Participants were assured that their participation or non-participation would not affect their clinical care. This study was approved by the Ethics Committee of the researchers’ institution and the participating hospital.

## Results

**Fig. 1:**
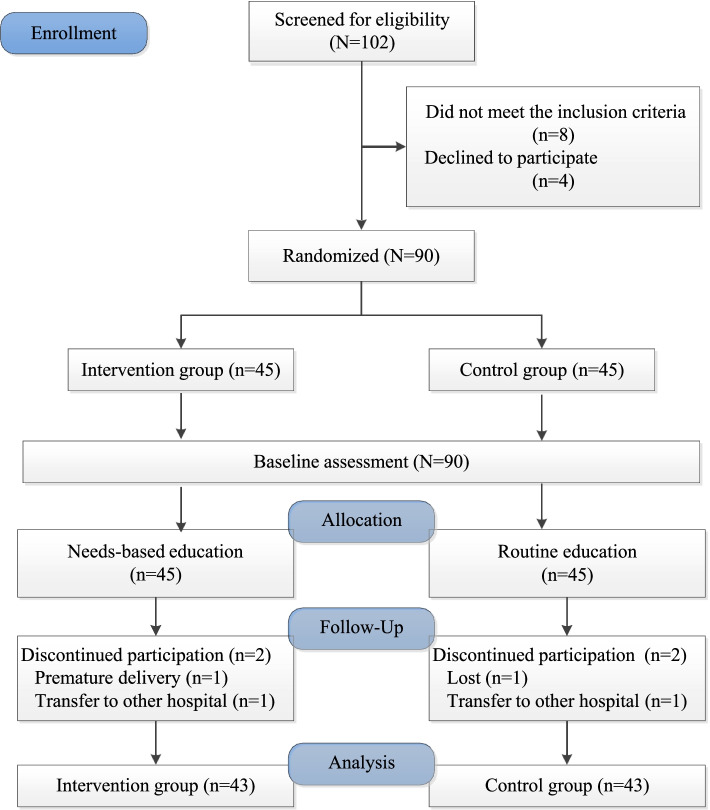
Flow diagram of recruitment and grouping

There were 102 women screened from the prenatal clinic from February to June 2021. According to the selection criteria, 94 women were eligible for inclusion in the study, and 90 (88.23%) agreed to participate and were enrolled. A total of 90 participants were randomly assigned to the intervention group (*n* = 45) and the control group (*n* = 45). Of the 90 enrolled participants, 86 (84.31%) completed the study, with 43 in the intervention group and 43 in the control group (see Fig. 1). There were no significant differences in socio-demographic characteristics between those followed up and those lost to follow-up (*P* > 0.05).

### Socio-demographic characteristics of participants

There were 86 advanced multiparas in the current study whose ages ranged from 35 to 44 years (36.95 years on average). See Table 2. There were no significant statistical differences in socio-demographic characteristics between the two groups.

**Table 2:** Socio-demographic characteristics of participants (*N* = 86)

Items	Total	Intervention group(***n*** = 43)	Control group(***n*** = 43)	Statistic	***P***
Age (years)	36.95 ± 2.25	36.95 ± 2.17	36.95 ± 2.36	0.30^a^	0.768
Weeks of gestation	27.01 ± 0.94	26.95 ± 0.95	27.07 ± 0.94	0.57^a^	0.567
Education level				0.05^b^	0.958
Junior school	3 (3.49)	2 (4.65)	1 (2.33)		
High school	12 (13.95)	6 (13.95)	6 (13.95)		
College and above	71 (82.56)	35 (81.40)	36 (83.72)		
Monthly income				0.93^b^	0.354
<1000	1 (1.16)	0 (0.00)	1 (2.33)		
1000–4999	15 (17.44)	7 (16.28)	8 (18.60)		
5000–9999	36 (41.86)	17 (39.53)	19 (44.19)		
>10,000	34 (39.54)	19 (44.19)	15 (34.88)		
Current residence				0.12^b^	0.725
Urban	77 (89.53)	39 (90.70)	38 (88.37)		
Rural	9 (10.47)	4 (9.30)	5 (11.63)		
Employment				0.45 ^c^	0.549
Yes	74 (86.05)	36 (83.72)	38 (88.37)		
No	12 (13.95)	7 (16.28)	5 (11.63)		
Family status				0.98^c^	0.323
Only child	22 (25.58)	9 (20.93)	13 (30.23)		
With siblings	64 (74.42)	34 (79.07)	30 (69.77)		
Gravidity				1.08^c^	0.279
2	49 (56.98)	27 (62.79)	22 (51.16)		
≥ 3	37 (43.02)	16 (37.21)	21 (48.84)		
Parity				0.84^b^	0.400
1	80 (93.02)	41 (95.35)	39 (90.70)		
2	6 (6.98)	2 (4.65)	4 (9.30)		
Previous delivery mode				0.05^c^	0.829
Vaginal	39 (45.35)	20 (46.51)	19 (44.19)		
Caesarean section	47 (54.65)	23 (53.49)	24 (55.81)		
Planned pregnancy				1.33^c^	0.516
Planned	64 (74.42)	33 (76.74)	31 (72.09)		
Accidental	22 (25.58)	10 (23.26)	12 (27.91)		

^a^ two sample *t* test

^b^ Fisher’s exact test

^c^ Chi-square test

### Comparison of pregnancy-related anxiety questionnaire scores

There was no statistically significant difference between PAQ scores of the two groups at baseline (*t* = − 0.63, *P* > 0.05), as shown in Table 3. After the intervention, PAQ scores of the intervention group were significantly lower than at baseline and those of the control group (*t* = 8.01, *P* < 0.05; *t* = 4.21, *P* < 0.05). No significant differences were found between the pre-test and post-test PAQ scores of the control group (*t* = 1.04, *P* > 0.05).

**Table 3:** Comparisons of pregnancy-related anxiety questionnaire and sub-scale scores

Scores	Intervention group (***n*** = 43)				Control group (***n*** = 43)				Pre-test		Post-test	
	Pre-test	Post-test	***t***	***P***	Pre-test	Post-test	***t***	***P***	***t***	***P***	***t***	***P***
PAQ	22.42 ± 4.42	19.28 ± 2.74	8.01^a^	< 0.001	21.86 ± 3.75	21.07 ± 3.38	1.04^a^	0.304	−0.63^b^	0.530	4.21^b^	< 0.001
anxiety of self	8.56 ± 1.70	8.93 ± 1.33	−2.28^a^	0.028	7.91 ± 1.63	9.14 ± 1.70	−13.23^a^	< 0.001	−1.94^b^	0.053	0.64^b^	0.527
anxiety of child health	9.76 ± 2.91	6.27 ± 1.26	9.47^a^	< 0.001	9.93 ± 2.40	8.34 ± 2.00	9.11^a^	< 0.001	0.61^b^	0.544	5.75^b^	< 0.001
anxiety of giving birth	4.09 ± 1.17	4.07 ± 0.94	0.24^a^	0.812	4.02 ± 1.22	4.58 ± 0.93	−4.60^a^	< 0.001	0.00^b^	0.996	2.54^b^	0.013

PAQ refers to the Pregnancy-related Anxiety Questionnaire

^a^ Paired sample t test

^b^ Two sample t test

Related to the three PAQ subscale scores, there were no statistically significant differences between the two groups at baseline (*t* = − 1.94, *P* > 0.05; *t* = 0.61, *P* > 0.05; *t* = 0.00, *P* > 0.05). After the intervention, the scores on ‘anxiety of child health’ and ‘anxiety of giving birth’ in the intervention group were statistically lower than those of the control group (*t* = 5.75, *P* < 0.05; *t* = 2.54, *P* < 0.05), whereas no significant difference was found in the scores on ‘anxiety of self’ between the two groups (*t* = 0.64, *P* > 0.05). Regarding within-group effects, the post-test scores of the intervention group on ‘anxiety of self’ and ‘anxiety of child health’ were significantly less than those at baseline (*t* = − 2.28, *P* < 0.05; *t* = 9.47, *P* < 0.05), whereas the ‘anxiety of giving birth’ did not indicate a significant difference (*t* = 0.24, *P* > 0.05). The post-test scores of the control group on ‘anxiety of child health’ were less statistically significant than those at baseline (*t* = 9.11, *P* < 0.05), whereas ‘anxiety of self’ and ‘anxiety of giving birth’ had statistically higher scores than at baseline (*t* = − 13.23, *P* < 0.05; *t* = − 4.60, *P* < 0.05) (see Table 3).

### Comparison of blood pressure and heart rate

At baseline there were no statistically significant differences in systolic blood pressure (SBP) or diastolic blood pressure (DBP) and HR between the two groups (*t* = − 0.18, *P* > 0.05; *t* = 0.07, *P* > 0.05; *t* = − 0.23, *P* > 0.05). The gestational ages at which post-test BP and HR were measured were 34.59 ± 1.19 (range: 33–36 weeks), and 34.84 ± 1.17 in the intervention group and 34.37 ± 1.18 in the control group, respectively. The post-test SBP and HR in the intervention group were significantly lower than those of the control group (*t* = 3.64, *P* < 0.05; *t* = 2.39, *P* < 0.05), while the DBP did not show any significant difference (*t* = 0.44, *P* > 0.05) (see Table 4).

**Table 4:** Comparisons of blood pressure and heart rate

Parameters	Intervention group (***n*** = 43)				Control group (***n*** = 43)				Pre-test		Post-test	
	Pre-test	Post-test	***t***	***P***	Pre-test	Post-test	***t***	***P***	***t***	***P***	***t***	***P***
SBP	113.23 ± 9.28	108.91 ± 10.59	2.06^a^	0.04	112.85 ± 10.08	116.75 ± 9.34	−1.86^a^	0.06	−0.18^b^	0.791	3.64^b^	<0.001
DBP	72.15 ± 7.70	76.79 ± 8.48	−2.66^a^	0.009	72.27 ± 7.14	77.60 ± 8.29	−3.19^a^	0.002	0.07^b^	0.995	0.44^b^	0.691
HR	79.06 ± 8.86	75.09 ± 9.30	2.04^a^	0.04	78.64 ± 7.80	80.23 ± 10.39	−0.80^a^	0.42	−0.23^b^	0.819	2.39^b^	0.016

SBP, DEP and HR refer to the systolic blood pressure, diastolic blood pressure and heart rate, respectively

^a^ Paired sample t test

^b^ Two sample t test

Regarding within-group effects, a significant decrease was found in SBP and HR in the intervention group (*t* = 2.06, *P* < 0.05; *t* = 2.04, *P* < 0.05), whereas the DBP was significantly increased (*t* = − 2.66, *P* < 0.05). In the control group, the post-test SBP, DBP and HR were increased from baseline (*t* = − 1.86, *P* > 0.05; *t* = − 0.80, *P* > 0.05), but only the DBP was statistically significant (*t* = − 3.19, *P* < 0.05).

### Comparison of non-stress test

At baseline, 39.53% of participants in the intervention group and 34.88% of participants in the control group had reactive NST results, and no significant difference was observed in the reactive NST results between the two groups (*X*^*2*^ = 0.58, *P* > 0.05). After the intervention, 58.14% of participants in the intervention group and 41.86% of participants in the control group had reactive NST results. However, no significant difference was found in the reactive NST results between the two groups (*X*^*2*^ = 2.39, *P* > 0.05).

## Discussion

The current study illustrated the effectiveness of needs-based education tailored for advanced multiparas on improving their prenatal anxiety. Expectedly, women who received needs-based education reported less anxiety related to the birth experience and their child’s health. The interventions also resulted in significant improvements in some physiological parameters, including SBP and HR.

The hypothesized effectiveness of needs-based education on prenatal anxiety was confirmed in the current study. This is in line with the previous studies using antenatal education to reduce pregnancy anxiety or related mental disorder which showed that insufficient knowledge of pregnancy may lead to anxiety [23, 24]. Women who received antenatal education increased their level of maternal knowledge, thus decreasing their level of anxiety. However, a qualitative study reported that antenatal education had different effects on anxiety, reducing or increasing the level of anxiety in pregnant women [25]. Given that the provision of knowledge about pregnancy may lead to anxiety [23], the education program was revised and characterized by targeting the pregnant women’s needs. The finding suggested that the current education program was effective by taking the individual’s/group’s needs into account. Specifically, as reported by previous studies [26–29], the greatest concerns for advanced multiparas in the current study included those of maternal age-related risks, medication use during pregnancy and psychological relaxation strategies. Therefore, the current education program targeting the greatest concerns in advanced multiparas might be helpful to satisfy the knowledge needs, sense of security, as well as the decreased general uncertainty of advanced multiparas directly, thereby, reassuring the level of anxiety in pregnant women [30].

As expected, the hypothesis concerning needs-based education in improving BP and HR was also confirmed. Three possible reasons could be taken into consideration to explain the result. Since they are anxiety-related physiological parameters, the BP and HR were associated with the fluctuation in anxiety. The decreased anxiety level indicates a relatively stable BP and HR. Another consideration might be the content of needs-based education. The progressive muscle relaxation training provided in the education program demonstrated a relationship with sympathetic nervous system activity alterations that could lower BP and HR [31]. However, the present study found that needs-based education was not able to improve the DBP and NST, and the reason may be due to a small sample size. In addition, the needs-based education intervention may also serve as an effective and safe way to lower BP in hypertensive disorders of pregnancy.

### Strengths and limitations

To the authors’ knowledge, this is the first study to present a needs-based education intervention program in the prenatal period, as well as in a vulnerable population (advanced multiparas). Moreover, needs-based education provides an innovative and interactive teaching strategy. Participants in the intervention group were highly motivated, actively participated and responded positively. Needs-based education also required less time, as the majority of participants were employed and had time constraints regarding their participation in education programs, which resulted in a succinct and focused approach by the researchers. In addition, objective indicators (e.g., BP, HR) were also used to provide evidence of the decreased level of anxiety.

However, limitations should be acknowledged. Sampling undertaken at a single hospital in central China limits the generalizability of findings to other geographic areas. Another limitation is that participants were mainly college-educated women and had moderately high-income levels, which might yield a sampling bias and limit the interpretation of the results. Moreover, although the sample size was sufficient to reach statistical significance related to prenatal anxiety, the study did not show differences in other parameters such as the DBP and NST. Future studies using a larger sample size are required to verify these results. In addition, although the two groups were comparable in terms of socio-demographic characteristics, some potential confounders may still exist in the current manuscript. Future research studies on the topic of prenatal anxiety should fully investigate and elaborate on its influencing factors such as smoking and physical activity habits.

## Conclusions

It can be concluded that needs-based education was effective in relieving prenatal anxiety as well as in improving some anxiety-related physiological parameters. Based on the findings of the current study, needs-based education is an effective approach to providing information to help pregnant women manage prenatal anxiety and can be incorporated into routine antenatal care.

## Data Availability

The datasets generated and/or analyzed during the current study are not publicly available due to privacy reasons but are available from the corresponding author upon reasonable request.

## Abbreviations

PAQ: Pregnancy-related Anxiety Questionnaire
BP: Blood pressure
DBP: Diastolic blood pressure
SBP: Systolic blood pressure
HR: Heart rate
NST: Non-stress test
